# Cheating on the Edge

**DOI:** 10.1371/journal.pone.0002763

**Published:** 2008-07-23

**Authors:** Lee Alan Dugatkin, Aaron D. Dugatkin, Ronald M. Atlas, Michael H. Perlin

**Affiliations:** 1 Department of Biology, University of Louisville, Louisville, Kentucky, United States of America; 2 Murray Hill Academy, Louisville, Louisville, Kentucky, United States of America; Oxford University, United Kingdom

## Abstract

We present the results of an individual agent-based model of antibiotic resistance in bacteria. Our model examines antibiotic resistance when two strategies exist: “producers”–who secrete a substance that breaks down antibiotics–and nonproducers (“cheats”) who do not secrete, or carry the machinery associated with secretion. The model allows for populations of up to 10,000, in which bacteria are affected by their nearest neighbors, and we assume cheaters die when there are no producers in their neighborhood. Each of 10,000 slots on our grid (a torus) could be occupied by a producer or a nonproducer, or could (temporarily) be unoccupied. The most surprising and dramatic result we uncovered is that when producers and nonproducers coexist at equilibrium, nonproducers are almost always found on the edges of clusters of producers.

## Introduction

The evolution of traits that may benefit others, as well as self, has long been an interest of evolutionary biologists [1]–[8]. This issue has been brought to the forefront recently by experimental work on group-beneficial traits in model bacterial and yeast systems [6], [9]–[20] including, but not limited to, work using *Staphylococcus aureus*, *Pseudomonas fluorescens, Pseudomonas aeruginosa, Myxococcus xanthus, Saccharomyces cerevisiae and Escherichia coli* **.** In addition, theoreticians have actively debated the role of individual, kin, group, and frequency-dependent selection in explaining the results of these experiments [6], [17].

Using both theoretical and empirical tools, we have been examining the evolution of group-beneficial traits in the context of bacterial antibiotic resistance in *E.coli.* In two earlier papers, we examined the evolution of “producers” (who secrete a substance that breaks down antibiotics) and nonproducers (“cheats”) who do not secrete, or carry the machinery associated with secretion. Our prior models examined the evolution of these strategies in a single, very large population [21], as well as in metapopulations containing discrete trait groups [22].

Here, we examine the evolution of producers and nonproducers using an individual agent-based model. The model allows for populations of up to10,000, in which bacteria are affected by their nearest neighbors. Each of the 10,000 slots on our grid (a torus) could be occupied by a producer or a nonproducer, or could (temporarily) be unoccupied. Our empirical work suggests that this agent-based model may best mimic the dynamics of bacterial interactions in the context of shared antibiotic resistance. For example, our experimental work has found that when β-lactamase is produced to break down antibiotics, it is tethered to the producer cell, and hence primarily affects the producer's nearest neighbors. Our agent-based model captures this dynamic in ways that prior models have not.

### The Model

We consider two genotypes, labeled producers and nonproducers. Producers create a substance that provides them with a benefit and provides benefits to other group members as well, while nonproducers do not produce such a substance. In this model the “substances” we focus on are enzymes, such as β-lactamase, that break down β-lactam antibiotics (e.g.,ampicillin). In terms of bacterial antibiotic resistance, producers will possess a gene (often, but not exclusively, plasmid-borne) that codes for an antibiotic resistance mechanism that protects them from damage due to antibiotics. Plasmid possession carries a cost, in that cellular resources are required for plasmid replication and maintenance [13]. Nonproducers do not carry the plasmid with the gene for antibiotic resistance, but receive protection as a function of the number of producers in their neighborhood (it is in that sense that we consider producers as providing group- or neighborhood-level benefits to others). If nonproducers are surrounded by other nonproducers they die (details below), and hence the typical “invasion” problems associated with group-beneficial traits do not apply to our producer strategy, as pure populations of nonproducers are not viable.

In our model, the benefit (B) associated with β-lactamase ranged from 0 to 1. Producers always received this benefit. Because β-lactamase may be “tethered” to the outside of a cell, we created a variable called “help” that measures the proportional benefit that cells near a producer receive, as a result of the β-lactamase tethered to that producer (that is; 0<help<B). Producers pay a cost (0<C<1) associated with β-lactamase production [13].

The fitness of the producers = **B−C+(number of producers in neighborhood x B x help)**

The fitness of the nonproducers

 = **0**; if no producers are in neighborhood

or

 = **number of producers in neighborhood x B x help**; if one or more producers are in neighborhood.

We used NetLogo simulation software [23] to build an agent-based model for the evolution of antibiotic resistance when producers and nonproducers interact. A 100×100 torus (no edges) with 10,000 “slots” was created, and we assumed that an antibiotic, such as ampicillin, was present at all times during our simulations. At the start of a simulation, each slot held either a producer or a nonproducer with probability 0.5 (qualitatively similar results were found when simulations were initiated with producer/nonproducer proportions of .20/.80, .30/.70, .40/.60, .60/.40, .70/.30, and .80/.20).

Neighborhood size was set at eight individuals–the so-called “Moore neighborhood”–corresponding to the eight slots that could be reached in a single move of a chess king [24]. Fitness values from generation 1 were calculated and used to populate the torus generation 2. If a slot was filled with a nonproducer, and its Moore neighbors were all nonproducers, that nonprodcuer died, and that slot was empty in generation 2. Otherwise, the fitness of the individual in a slot was added to the fitness of the individuals in its Moore neighborhood. The probability that a slot was occupied by a producer in generation 2 was simply the sum of all producer fitnesses in the Moore neighborhood over the total fitness in that neighborhood. Slots were filled in exactly the same way in each subsequent generation, with one exception: in generation 3 and beyond, if a slot was filled by a nonproducer and its Moore neighbors were nonproducers *or* empty slots, that slot was empty in the next generation.

Although any stochastic simulation will eventually settle at a homogenous absorbing state [24], [25], our pilot work found similar results when simulations were run for 1000, 5000 or 20,000 generations; hence we report results from 1000 generations.

## Results

**Figure 1:**
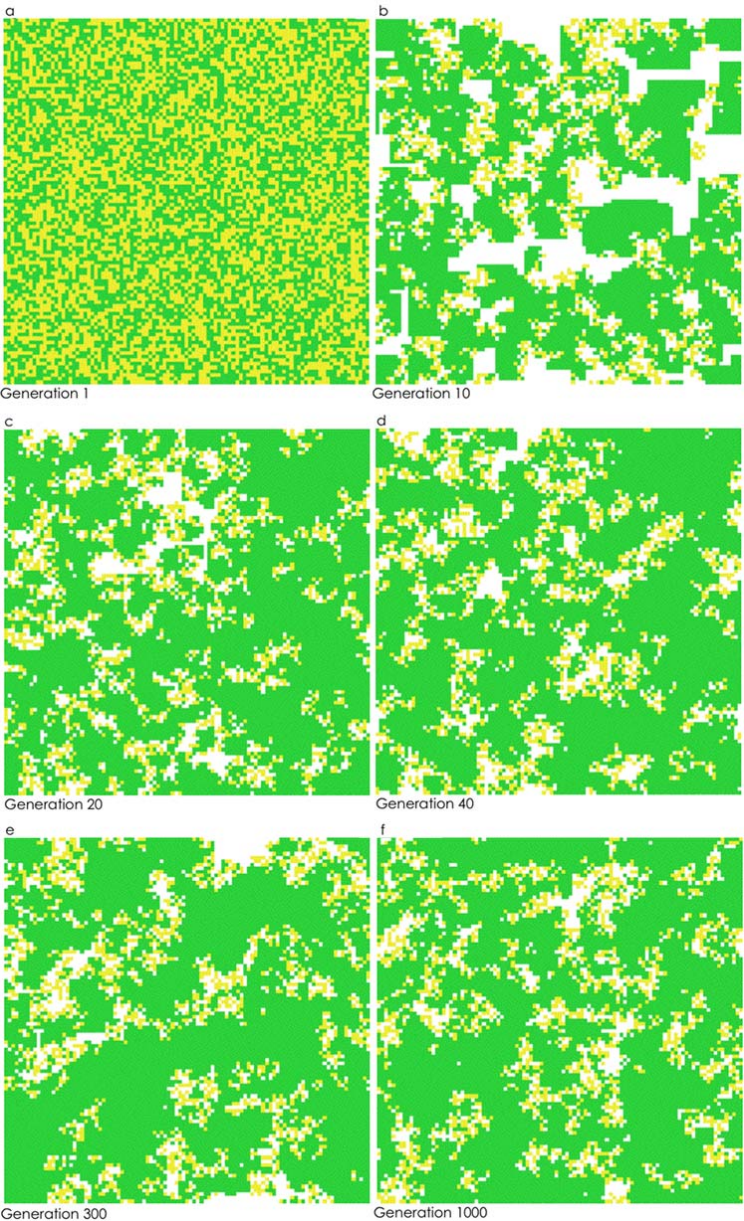
Two-dimensional snapshots of the 10,000 slot torus. B/C = 0.62, help = 0.14 (this “help” value was chosen, in part, as the result of unpublished experimental work on β–lactamase secretion in producer cells). a) Generation 1, b) Generation 10, c) Generation 20, d) Generation 40, e) Generation 300 and f) Generation 1000. Note that for generations 1–999, any yellow (nonproducers) cells surrounded by only yellow or by only yellow and white cells would die and be replaced the next generation.

**Figure 2:**
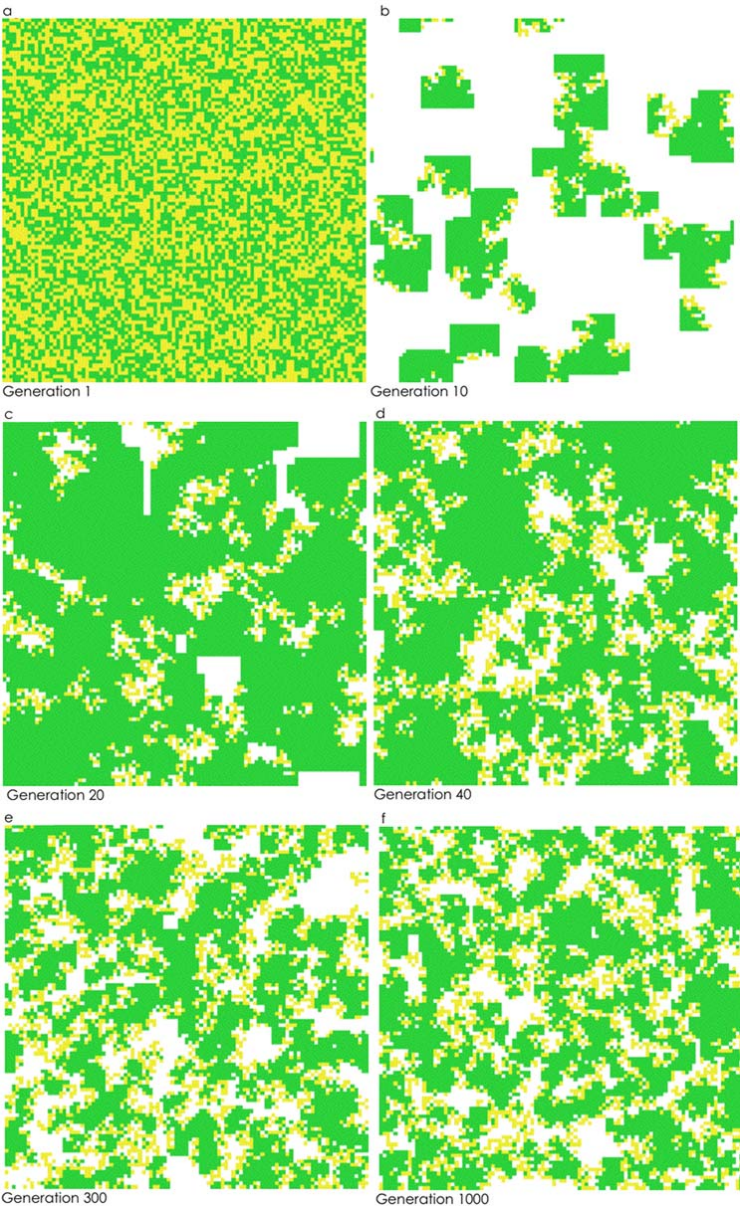
Two-dimensional snapshots of the 10,000 slot torus. B/C = 0.56, help = 0.19. a) Generation 1, b) Generation 10, c) Generation 20, d) Generation 40, e) Generation 300 and f) Generation 1000. Note that for generations 1–999, any yellow (nonproducers) cells surrounded by only yellow or by only yellow and white cells would die and be replaced the next generation.

**Figure 3:**
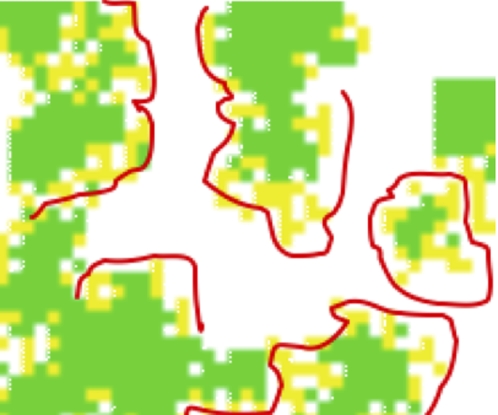
Zoomed-in version of upper left hand section of a snapshot at generation 1000. B/C = 0.62, help = 0.09. Red lines outline some cases in which nonproducers exist at the boundary between empty space and clusters of altruists. In all figures, yellow = nonproducers, green = producer, white = empty slot.

We examined thirty B/C scenarios from rations of 2∶1 down to 0.5∶1, as well as six different values for “help.” When B>C, producers went to fixation very quickly in our model. When C>B, we found, not unexpectedly, that increasing the B/C ratio increased the equilibrial frequency of producers. In addition, increasing the value of “help” increased the frequency of producers. Our most novel, and potentially important, finding was that if C>B, and producers and nonproducers coexisted at equilibrium, nonproducers were almost always found on the edge (boundary) of clusters of producers; more specifically, nonproducers were found between clusters of producers and areas of open space (i.e., open slots; Figures 1, 2 and 3). Across all simulations where producers and nonproducers were present at generation 1000, a producer typically had about seven other producers in its Moore neighborhood (and 0.5 empty cells plus 0.5 nonproducers); a nonproducer usually had just two nonproducers, about three producers, and three empty cells in its neighborhood.

Although the exact positioning of producers, nonproducers and empty slots constantly changed over the course of a thousand generations, this spatial patterning, in which producers are found on the edge between clusters of producers and empty slots, was similar across generations.

## Discussion

There is a large and rapidly growing literature on microbes as model systems to test “public goods” problems (see references in Introduction). The results of our model are in agreement with some general findings from this work: for example, as the B/C ratio increases, cooperation increases. Our model uncovered an interesting, novel spatial patterning for producers and nonproducers, wherein nonproducers–the equivalent of cheats in our simulations–were almost always found on the boundary between clusters of producers and empty (unoccupied) slots. This was in part a result of the strong frequency-dependent nature of the payoffs in the model, especially the harsh payoff when cheaters were surrounded by other cheaters. Though the position of producers, nonproducers and empty slots moved around through time, the “cheaters-on-the-edge” phenomenon moved with it. This dramatic spatial pattern appears to be the result of the following dynamics: when surrounded by other nonproducers or by empty space, nonproducers in our model die (due to antibiotics). What that means is that clusters of nonproducers perish, and nonproducers cannot survive in the middle of “clusters” of empty slots. When nonproducers find themselves in the midst of clusters of producers, they do poorly because of the dynamics of competition at the local scale. For example, consider the case of a nonproducer whose Moore neighbors are all producers. The relative fitness of that nonproducer is higher than any of its producer neighbors, but the particular strategy that will fill that nonproducer's slot in the next generation is a function of the fitness of producers and nonproducers in that Moore neighborhood *weighted by their prevalence in that neighborhood*, and hence the nonproducer slot is very likely to be taken by a producer in the next generation.

Why then do nonproducers survive on the moving boundaries between clusters of producers and areas of empty space? In these areas, nonproducers can often fill empty slots because they are closer to those empty slots than producers (who cluster with each other). But, nonproducers cannot continue to fill further empty slots because once they begin to cluster in formerly empty space, they die in the presence of antibiotics, when no producers are nearby. This introduces a temporal component to the results, wherein nonproducers appear to “chase” empty slots, and producers appear to chase the nonproducers.

The fact that producers clustered together quickly in our simulations also helps explain why increasing the value of “help” – the proportional benefit that cells near producers receive–increased the frequency of producers. Clustering created a situation in which producers were most often aiding others producers, and this, in conjunction with the phenomena outlined above, will allow us to better understand why providing greater levels of help differentially aided producers.

We recognize that our model examines only two strategies and that costs and benefits themselves may evolve: more work is needed on these fronts. Nevertheless, it is our hope that these results will spur empirical researchers working on group-beneficial traits in bacterial model systems to search for spatial and temporal patterns similar to those we have observed in our agent-based simulations. In terms of medical implications, because nonproducers in our model were sensitive to antibiotics, and were often found between empty spaces and producers, targeting such areas with antibiotic infusions may be worth considering.
